# One-pot synthesis of hierarchical FeZSM-5 zeolites from natural aluminosilicates for selective catalytic reduction of NO by NH_3_

**DOI:** 10.1038/srep09270

**Published:** 2015-03-20

**Authors:** Yuanyuan Yue, Haiyan Liu, Pei Yuan, Chengzhong Yu, Xiaojun Bao

**Affiliations:** 1 State Key Laboratory of Heavy Oil Processing, China University of Petroleum, Beijing 102249, P. R. China; 2 The Key Laboratory of Catalysis, China National Petroleum Corporation, China University of Petroleum, Beijing 102249, P. R. China; 3 Australian Institute for Bioengineering and Nanotechnology, the University of Queensland, Brisbane St Lucia, QLD 4072, Australia

## Abstract

Iron-modified ZSM-5 zeolites (FeZSM-5s) have been considered to be a promising catalyst system to reduce nitrogen oxide emissions, one of the most important global environmental issues, but their synthesis faces enormous economic and environmental challenges. Herein we report a cheap and green strategy to fabricate hierarchical FeZSM-5 zeolites from natural aluminosilicate minerals via a nanoscale depolymerization-reorganization method. Our strategy is featured by neither using any aluminum-, silicon-, or iron-containing inorganic chemical nor involving any mesoscale template and any post-synthetic modification. Compared with the conventional FeZSM-5 synthesized from inorganic chemicals with the similar Fe content, the resulting hierarchical FeZSM-5 with highly-dispersed iron species showed superior catalytic activity in the selective catalytic reduction of NO by NH_3_.

Iron modified zeolites, especially Fe-modified ZSM-5 zeolites (FeZSM-5s), have been reported to be potential and active catalysts for a number of reactions, including N_2_O decomposition^1^,^2^, selective catalytic reduction (SCR) of nitrogen oxides (NO_x_)^3^,^4^, selective oxidation of methane to methanol^5^,^6^, selective hydroxylation of benzene to phenol^7^,^8^ and some other important reactions^9^,^10^,^11^,^12^. Particularly, it is worth to note that SCR of NO_x_ by ammonia (NH_3_-SCR) over FeZSM-5s is considered to be the most attractive and effective route to removing NO_x_ from industrial off-gases and diesel engine exhausts that have been widely known as a major cause of photochemical smog, acid rain and ozone depletion^3^,^13^,^14^,^15^,^16^,^17^,^18^,^19^,^20^,^21^.

So far, different methods have been developed to prepare FeZSM-5s with desired physicochemical and catalytic properties, including wet ion exchange^10^,^22^,^23^, solid ion exchange^16^,^24^, chemical vapor deposition (CVD) of volatile iron compounds^25^,^26^ and isomorphous substitution methodology^27^,^28^. However, the large-scale utilization of exchanged catalysts in industry is not attractive because of their complex preparation process and worse reproducibility due to the formation of large iron-oxide particles during calcination which are widely accepted to be inactive in the different reactions catalyzed by FeZSM-5s^16^,^27^. While being suggested as a more reproducible method for preparing over-exchanged FeZSM-5s, CVD of FeCl_3_ in the channels of HZSM-5 always produces corrosive HCl gas which seriously damages the facilities and sometimes needs vacuum/nitrogen conditions in the treatment of the zeolite, also unsuitable for large-scale production^1^. Meanwhile, all of these approaches use synthetic aluminum-, silicon-, and iron-containing chemicals that are made from natural aluminosilicate/silicate minerals and iron ores through complicated processes associated with huge waste production and extensive energy consumption, which makes the whole process not green from the source, even targeting at a green application. Additionally, internal mass transfer limitations are observed in the NH_3_-SCR of NO_x_ over FeZSM-5s, although the molecular sizes of the reactants and products are much smaller than the channel dimension of FeZSM-5s^29^. A feasible alternative to circumvent this issue is the introduction of mesopores into the zeolite structure, yielding hierarchical zeolites containing both micropores and mesopores. In the last two decades, synthesis of hierarchical zeolites has drawn extensive attention due to their improved performance in overcoming diffusion limitations and thereby enhancing catalytic properties^8^,^28^. However, the existing methods for preparing hierarchical zeolites exclusively involve the use of various mesoscale templates (e.g. polystyrene^9^, organosilane^28^ and surfactants^7^,^8^) or post-synthesis modifications (e.g. steaming treatment^30^, acid leaching^31^ or alkali leaching^32^), which bring about great energy and environmental stresses. To break these predicaments, it is highly desirable to develop an economical and environmentally friendly strategy to synthesize hierarchical FeZSM-5s.

Recently, we attempted to synthsize zeolites Y and ZSM-5 from natural aluminosilicate minerals, and interestingly we found that most of iron existing in the minerals as an impurity element could be incorporated into the product zeolite^33^,^34^. Inspired by this finding, herein we first report a facile and green one-pot synthesis strategy to prepare hierarchical FeZSM-5s via the nanoscale depolymerization-reorganization of natural aluminosilicate minerals neither using any aluminum-, silicon-, or iron-containing chemical nor involving any mesoscale template and any post-treatment, as shown in Figure 1. The catalytic results indicate that the resultant FeZSM-5s exhibited superior physicochemical properties and outstanding catalytic performance for NH_3_-SCR of NO.

## Results and Discussion

### Depolymerization of the natural minerals

The natural silicon-rich diatomite and aluminum-rich rectorite minerals were depolymerized via thermal and submolten salt (SMS) treatment, respectively. As we previously reported^33^,^34^, the natural rectorite mineral could be efficiently depolymerized via such a SMS system and the depolymerization temperature was 250°C that is far below the temperature of conventional thermal treatment (*ca*. 800°C). Additionly, the silicon and aluminum species in the SMS depolymerized rectorite exhibited high reactivity and were ideal nutrients for zeolite synthesis, and therefore the SMS depolymerization method can be really regarded as a green one.

### Synthesis of FeZSM-5s

In our approach, we employed the thermally activated diatomites of different grades and the SMS depolymerized rectorite as the sole silicon and aluminum sources for synthesizing hierarchical FeZSM-5s, and because all of the minerals have iron impurity, they are also explored as the iron source simultaneously. A series of FeZSM-5s with different iron contents were hydrothermally synthesized using tetrapropylammonium bromide (TPABr) as the single micropore template in the synthesis system. For a typical synthesis, the molar composition of the mixture was Al_2_O_3_:Fe_2_O_3_:SiO_2_:Na_2_O:TPABr:H_2_O = 1.1:0.26:40:6:4:1600. The resultant Fe-containing hierarchical ZSM-5 zeolite was named as FeZ-DR. For comparison, a reference FeZSM-5 zeolite denoted as FeZ-CA was also prepared by using water glass, sodium aluminate, iron nitrate and TPABr as silicon, alumina and iron sources and template, respectively, under the same conditions used for synthesizing FeZ-DR.

### Crystalline structure

**Figure 2:**
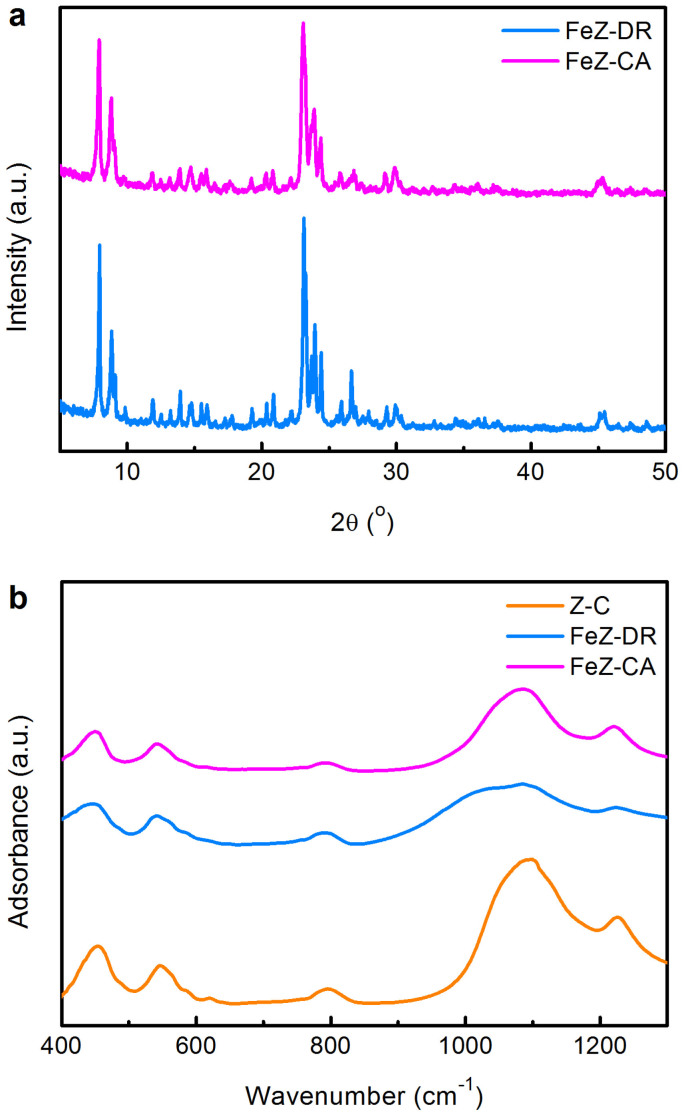
Crystalline structure. (a) XRD patterns and (b) FT-IR spectra of the different zeolites.

Figure 2 shows the X-ray diffraction (XRD) patterns and Fourier transform infrared (FTIR) spectra of FeZ-DR and FeZ-CA. As shown in Figure 2a, there is no obvious difference between the XRD patterns of FeZ-DR and FeZ-CA. Both of the two samples exhibit characteristic diffraction peaks appearing at 2θ = 7.9°, 8.8°, 23.1°, 23.9°, and 24.4°, which are exclusively indexed to the structure of MFI topology, and no reflection ascribed to Fe_2_O_3_ phase is observed, indicating the high crystallinity (90% for FeZ-DR and 86% for FeZ-CA) and purity of the samples. The crystallinity of the samples was also assessed from the intensity ratio of the vibration band at 550 cm^−1^ over that at 450 cm^−1^ in the FTIR spectroscopy^28^,^35^. The existence of the 550 cm^−1^ band is due to the asymmetric stretching of the double five-numbered ring of MFI zeolite, and the vibrational band at 450 cm^−1^ is assigned to T-O (where T denotes Si, Al or Fe) bending vibrations^36^,^37^,^38^. The relative crystallinity of FeZ-DR calculated from the FTIR data is slightly higher (97%) than that of FeZ-CA (94%), consistent with the results estimated from the XRD characterizations.

Figure 2b also shows that the most intense band at 1100 cm^−1^ attributed to the asymmetric stretching of T-O bond shifts to lower frequency for FeZ-DR and FeZ-CA (1083 and 1086 cm^−1^, respectively) as compared to that of a commercial Fe-free ZSM-5 (Z-C, purchased from Nankai University Catalyst Company, Tianjin, P. R. China). This shift can be interpreted as the longer Fe-O bond distance (1.84 Å) as compared to the Al-O bond distance (1.75 Å), strongly suggesting the substitution of Fe in the zeolite framework^39^. Flanigen *et al.*^40^ found that the shift in the main asymmetric band towards higher frequency on substitution of P in the zeolite framework is because of the shorter tetrahedral P-O bond distance (1.54 Å), well in agreement with our result. In addition, we also observed that both FeZ-DR and FeZ-CA contain a certain amount of Fe according to the chemical composition analysis data given in Table 1. From the XRD, FTIR and chemical composition analysis results, we can safely draw a conclusion that the above two samples synthesized are iron-containing ZSM-5 zeolites. It must be pointed that neither FeZ-DR nor FeZ-CA shows any XRD peaks corresponding to aggregated iron oxide species, indicating that the Fe species in the two zeolites are well dispersed or framework-incorporated^7^.

### Nature and distribution of iron species

**Figure 3:**
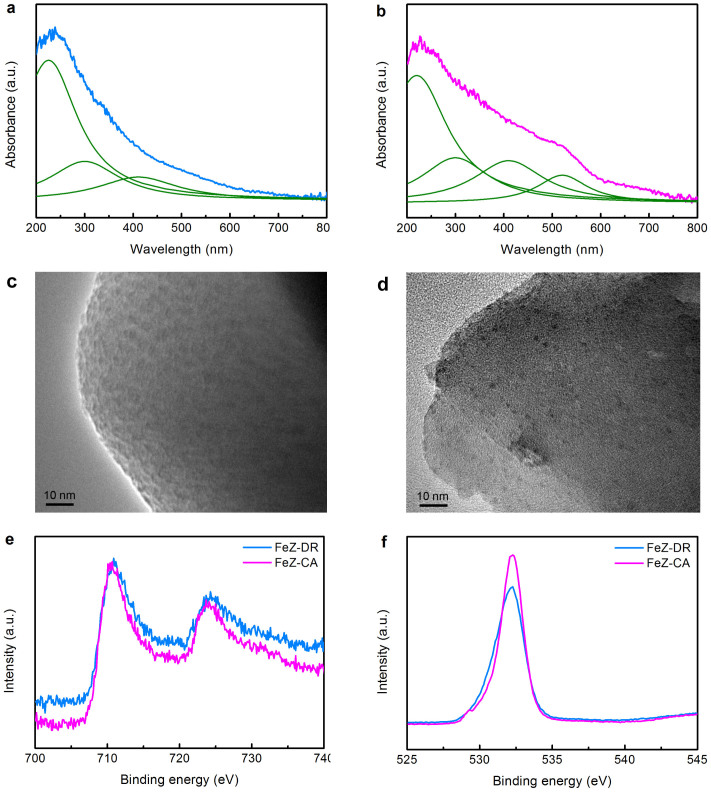
Nature and distribution of iron species. UV-visible spectra and TEM images of (a, c) FeZ-DR and (b, d) FeZ-CA and XPS spectra of (e) Fe 2p and (f) O 1s regions for FeZ-DR and FeZ-CA.

To identify the nature and distribution of the ferric ions in FeZ-DR and FeZ-CA, UV-visible spectroscopy, transmission electron microscopy (TEM) and X-ray photoelectron spectroscopy (XPS) characterizations of the samples were conducted and the results are shown in Figure 3. As seen in Fig. 3a and 3b, both the UV-visible spectra have a dominant absorbance band at ~225 nm due to the oxygen-to-iron charge transfer, indicating the existence of isolated Fe species which are introduced into the zeolite framework in tetrahedral coordination^8^,^41^. This is further confirmed by the FTIR and temperature programmed reduction (TPR) characterizations (Figure 2b and Supplementary Figure S1). As seen in Supplementary Figure S1, the H_2_-TPR profiles of FeZ-DR and FeZ-CA show a broad peak centered at *ca*. 730°C attributed to framework Fe (III) that is hard to reduce^27^,^42^. Additionally, the UV-visible absorption features in the region of 250–350 nm of the two samples are assigned to isolated and oligonuclear extra-framework Fe clusters in the zeolite channels, and those in the region of 350–450 nm are attributed to larger extra-framework Fe clusters^1^. The remarkable difference, however, is that FeZ-CA presents an additional UV-visible absorbance peak at ~520 nm, implying the existence of Fe_x_O_y_ nanoparticles on the external surface of zeolite crystals^41^. Here we infer that the Fe_x_O_y_ nanoparticles may be <~4 nm in size, since the XRD patterns show no characteristic peaks belonging to iron oxide species^7^ (Figure 2a). This is proved by the TEM images in Figure 3c and 3d. It is clearly seen that there is no dark spots on the external surface of FeZ-DR, while there are many dark spots (about 1 nm in size) on the external surface of FeZ-CA belonging to iron-containing particles^1^,^2^,^27^,^43^. It turns out that no iron species exist on the external surface of FeZ-DR, but many iron oxide particles of the size *ca*. 1 nm occur on the external surface of FeZ-CA, although the two samples own the identical Fe content (Table 1).

Deconvolution of the UV-visible absorbance bands into Gaussian subbands by following the standard fitting procedure gives the percentages of the different Fe species (Figure 3a and 3b and Table 2). The comparison for the same type of iron species in the different samples should not be influenced by the extinction coefficient because the positions of subbands used are the same for all of the samples. Therefore, this quantification does not account for the dependence of the extinction coefficient on the wavelength, but nevertheless provides a semi-quantitative estimation of the distributions of the various Fe species^44^. The results indicate that the percentage of the isolated framework Fe species in FeZ-DR is much higher than that in FeZ-CA that are the active sites for NH_3_-SCR of NO_x_^20^,^45^, and the fractions of the isolated, oligonuclear and larger Fe clusters in both zeolites are equal, which are active sites for many reactions such as N_2_O decomposition and benzene oxidation to phenol^7^,^10^; while FeZ-CA contains supererogatory 12% bulky iron oxide aggregates which are known to make no contribution to catalytic activity in the different reactions catalyzed by FeZSM-5s, such as NH_3_-SCR of NO_x_^27^. Moreover, in comparison with the results in the literature^1^,^7^, more iron species are incorporated into the zeolite framework via the new strategy proposed in this study.

XPS is a versatile surface analysis technique that can be used to qualitatively determine the ionic states of iron. Figure 3e shows the Fe 2p XPS results of FeZ-DR and FeZ-CA. Two peaks centered at ~711 and 725 eV corresponding to Fe 2p_3/2_ and Fe 2p_1/2_^18^,^39^, respectively, are clearly seen in the spectra. The Fe 2p_3/2_ peak is narrower and its intensity and area are higher than those of Fe 2p_1/2_ peak, which is due to the spin-orbit (j-j) coupling^18^. The obtained XPS spectra indicate that iron ions are in the trivalent oxidation state in both samples. Obviously, a small but notable difference between FeZ-DR and FeZ-CA in Figure 3f is that only one O 1s peak at ~532.2 eV corresponding to zeolite lattice oxygen is observed for FeZ-DR, while an additional peak centered at ~529.1 eV attributed to the oxygen in Fe_x_O_y_ is witnessed for FeZ-CA^39^. Similarly, Stencel *et al.*^46^ also found that there are two O 1s at 532 and 529 eV due to oxygen ions in the zeolite lattice and Fe_2_O_3_ in the iron-containing ZSM-5 zeolite sample, respectively, which is in line with the XPS results in the present research.

Based on the above UV-visible, H_2_-TPR, TEM and XPS characterization results, we can unequivocally conclude that the nature and distribution of the different Fe^3+^ species in FeZ-DR and FeZ-CA are different: in the former, only isolated framework Fe^3+^ species and isolated, oligonuclear and larger Fe extra-framework clusters are observed; whereas in the latter, in addition to the above three types of iron species, a fourth type of iron species Fe_2_O_3_ nanoparticles are detected. The reason for such difference is that the raw materials used for preparing FeZ-DR and FeZ-CA are entirely different. Unlike in inorganic Fe-containing chemicals such as iron nitrate, the iron species in the thermally activated diatomite and SMS depolymerized rectorite intrinsically have similar tetrahedrally coordinated structure compared to the fully crystallized framework iron sites in the Fe-zeolite (Supplementary Figure S2). According to chemical compositions of the mother liquor at different crystallization times (Supplementary Figure S3), the concentration of the ferric oxide in the mother liquor is always nearly zero, suggesting that the iron species in the minerals are transformed in situ into the product zeolite without experiencing the dissolution and incorporation steps as occured in using inorganic ferric salts (e.g. ferric nitrate) for synthesizing FeZ-CA. Therefore, a greater proportion of isolated Fe^3+^ in tetrahedral coordination is obtained in FeZ-DR.

### Hierarchical structure

**Figure 4:**
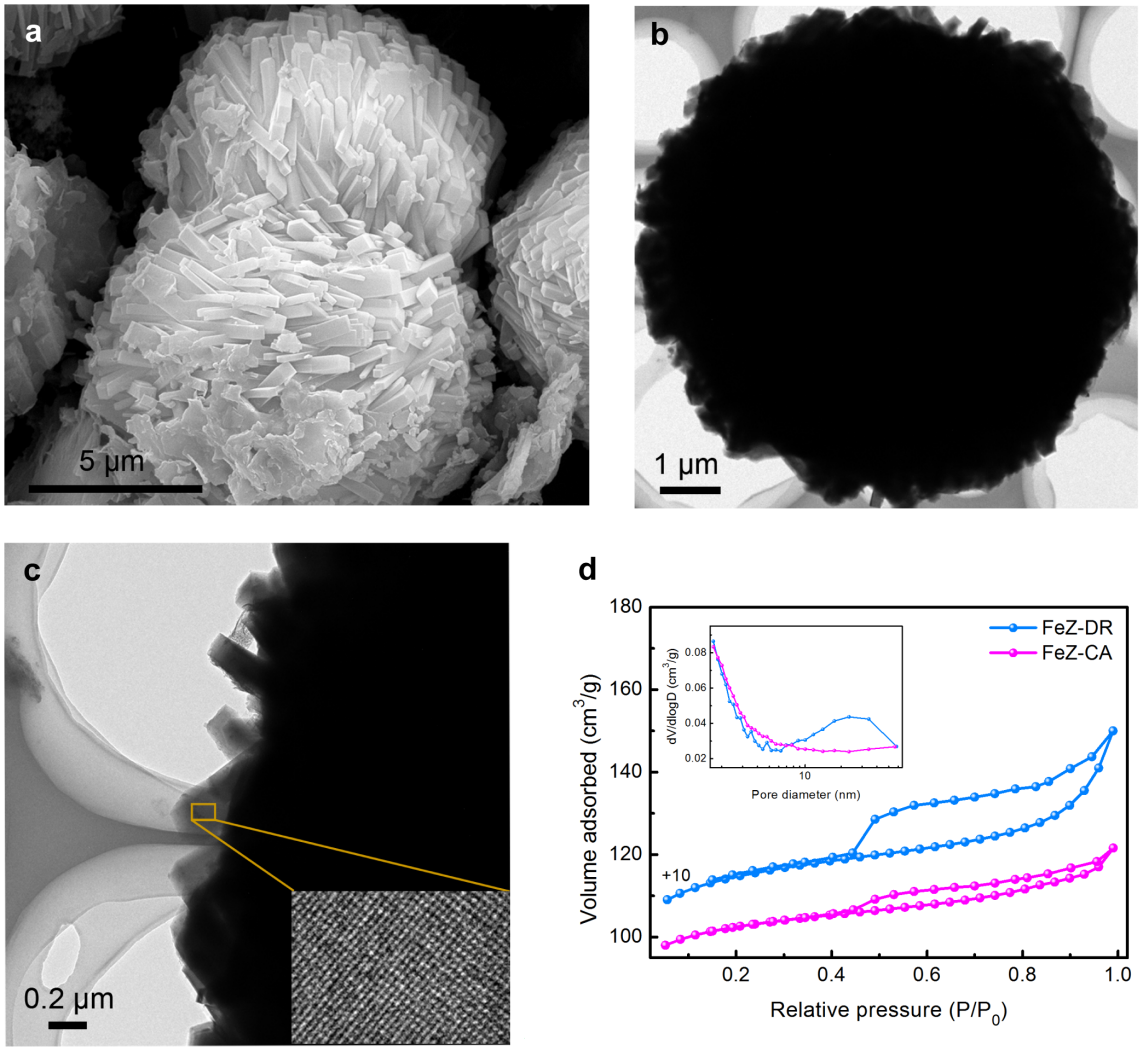
Hierarchical pore structure. (a) FESEM image of FeZ-DR; (b, c) TEM images of a single hierarchical FeZ-DR particle at low and high magnifications (the inset in c is the lattice fringes of FeZ-DR); and (d) nitrogen adsorption-desorption isotherms and BJH pore size distributions (inset) of FeZ-DR and FeZ-CA.

The field-emission scanning electron microscope (FESEM) and TEM images in Figure 4a–c clearly exhibit that FeZ-DR has a uniform spheroidal morphology formed from densely stacked nanorods, suggesting that it may have intercrystalline mesopores, i.e., interstitials among these nanorods^47^. It is worth mentioning that the lattice fringes can be clearly observed from the high resolution TEM (HRTEM) image (inset in Figure 4c), further corroborating the high crystallinity of FeZ-DR. Figure 4d shows the nitrogen adsorption-desorption isotherms and Barret-Joyner-Halenda (BJH) pore size distributions obtained from adsorption branches of the isotherms. The isotherms of FeZ-DR exhibit a significantly high uptake in the region P/P_0_ < 0.1 due to the presence of microporosity; interestingly, the isotherms of FeZ-DR show a predominant type IV shape with a large hysteresis loop at P/P_0_ > 0.4 that is typical for mesoporous materials, suggesting the presence of a considerable amount of mesopores^35^. The BJH pore size distribution clearly shows that FeZ-DR has a wide size distribution of mesopores of about 10 ~ 50 nm, attributed to the intercrystalline voids between those closely stacked claviform nanounits. Differently, the isotherms of FeZ-CA only display a small hysteresis loop and a steep N_2_ uptake at low relative pressure (P/P_0_ < 0.1), illustrating the predominant presence of microporous structure in the sample; significantly, the BJH profile shows no mesopore size distribution, revealing that FeZ-CA is a typical microporous material. The textural properties of FeZ-DR and FeZ-CA are given in Table 1. We can see that the former has much larger Brunauer-Emmett-Teller (BET) area, mesoporous area and mesoporous volume than the latter. This further declares that FeZ-DR possesses both microporous framework structure and abundant mesopores, which should benefit the catalytic reactions, especially those so-called diffusion-controlled reactions.

### Synthesis procedure

**Figure 5:**
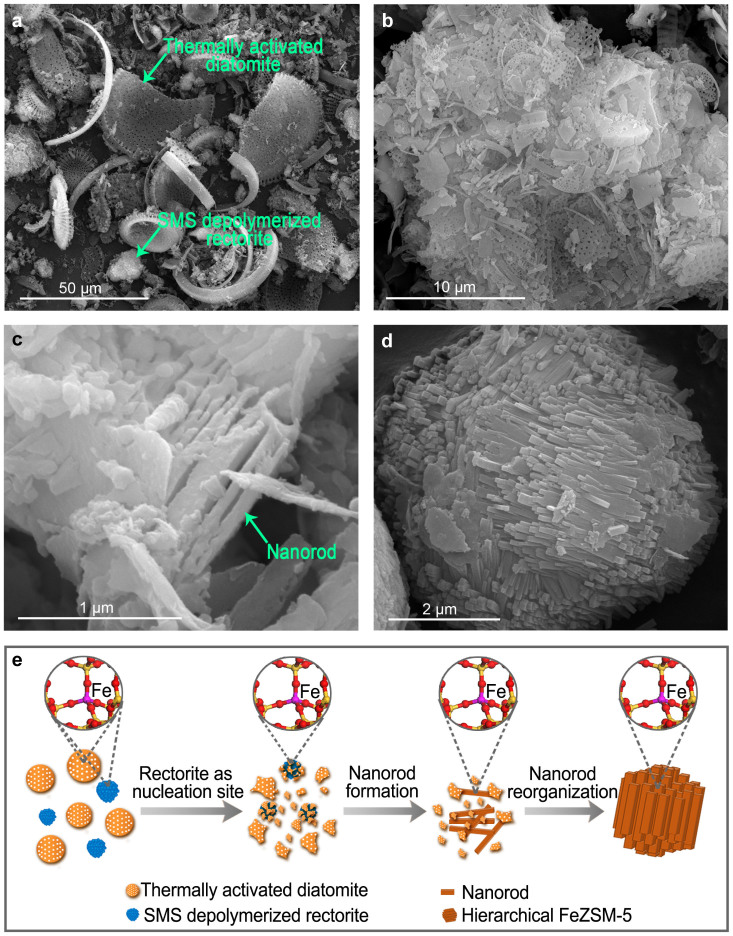
Synthesis process of FeZ-DR. (a–d) FESEM images of the solid samples at crystallization of 0, 4, 12, 48 h, respectively; and (e) schematic illustration of the formation process of FeZ-DR.

**Figure 6:**
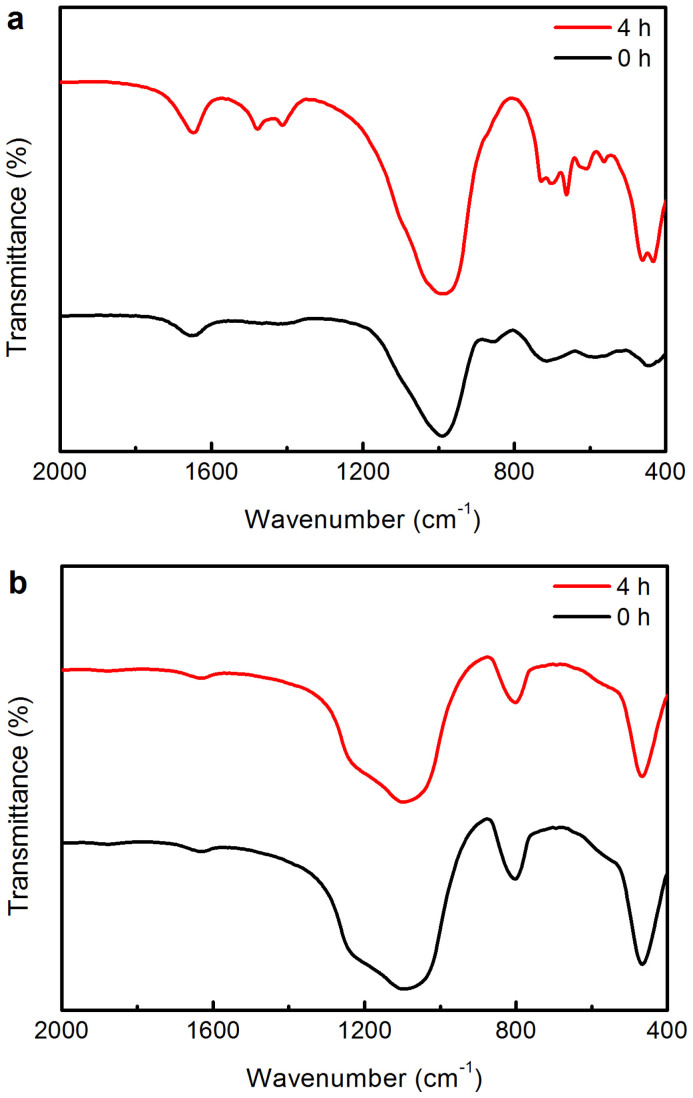
Characterizations of the different samples. FTIR spectra of the solid samples in (a) SMS depolymerized rectorite + TPABr and (b) thermally activated diatomite + TPABr systems at different crystallization times.

To get a better understanding of the synthesis, the crystallization process of FeZ-DR was carefully investigated by FESEM, as shown in Figure 5. Before crystallization, the image in Figure 5a shows that the sample is the mechanical mixture of the thermally activated diatomite and SMS depolymerized rectorite. After crystallization for 4 h, the thermally activated diatomite has already broken into plenty of smaller pieces in the alkaline environment and covers on the surface of the SMS depolymerized rectorite (Figure 5b). This is because the highly reactive silicon, aluminum and iron species in the SMS depolymerized rectorite can be transformed in situ into zeolitic precursors and act as crystalline seeds under the hydrothermal synthesis conditions^47^,^48^. This conclusion is supported by another experiment that the template TPABr reacted with the SMS depolymerized rectorite and thermally activated diatomite, respectively, and the results are shown in Figure 6. From Figure 6a, we can clearly see that some new bands at 1480 and 1410 cm^−1^, between 800 and 500 cm^−1^, and at 461 and 435 cm^−1^ are formed after crystallization for 4 h. The bands at 1480 and 1410 cm^−1^ are due to the CH_2_- bending vibration of TPA, suggesting that the SMS depolymerized rectorite has reacted with TPABr. The bands between 800 and 500 cm^−1^ are assigned to the tetrahedral vibrations formed by secondary building units and fragments of amorphous aluminosilicate network structure, the band at 461 cm^−1^ corresponds to the internal linkage vibrations due to the TO_4_ tetrahedra that are common to all zeolites and amorphous aluminosilicates^49^, and the band at 435 cm^−1^ is attributed to characteristic of five ring T-O-T^50^, indicating the presence of zeolitic precursors in the crystallization system^51^,^52^. However, there is no change in the thermally activated diatomite + TPABr system after crystallization for 4 h (Figure 6b), illustrating that there is no reactions occurring between the thermally activated diatomite and TPABr. This contrasting result shows that the TPABr reacts preferentially to the SMS depolymerization rectorite. Additionally, the crystallization curve of the SMS depolymerized rectorite-containing synthesis system (Supplementary Figure S4) shows a much faster nucleation rate and a shorter induction period, well in agreement with the earlier report on the synthesis of zeolite with the addition of crystalline seeds^47^,^48^. Therefore, when the SMS depolymerized rectorite is introduced into the synthesis system, a large amount of nuclei are formed; but when aluminum sulfate instead of the SMS depolymerized rectorite was used, only amorphous solid was obtained (Supplementary Figure S5).

Increasing the crystallization time to 12 h, nanorods have been observed in the solid sample (Figure 5c). Unlike inorganic silicon-containing chemicals (e.g. sodium silicate), the dissolution rate of the thermally activated diatomite in the sodium hydroxide (NaOH) solution is much slower; during crystallization, the concentrations of the active alumina and silica species are always at a lower level in the solution because of the employment of the thermally activated diatomite (Supplementary Figure S3), thus insufficient “nutrients” hinder the fast growth of primary nanorods already formed in the system into large single crystals but benefit the reorganization of them into hierarchical zeolites, as reported by Fang *et al.*^48^ Correspondingly, the aggregates composed by lots of densely stacked nanorods were observed (Figure 5d). Conversely, when sodium silicate instead of the thermally activated diatomite was used, a conventional microporous zeolite was obtained (Supplementary Figure S6). The whole crystallization process can be considered as an in situ zeolitization due to the relatively low concentrations of silicon, aluminum and iron species in the synthesis solutions at various times (Supplementary Figure S3), and thus more iron species are incorporated in situ into the zeolite framework (Supplementary Figure S7). From the above discussion, the reorganization procedure from the depolymerized minerals to the hierarchical FeZSM-5 zeolite is schematically illustrated in Figure 5e.

### Extension of the methodology

**Figure 7:**
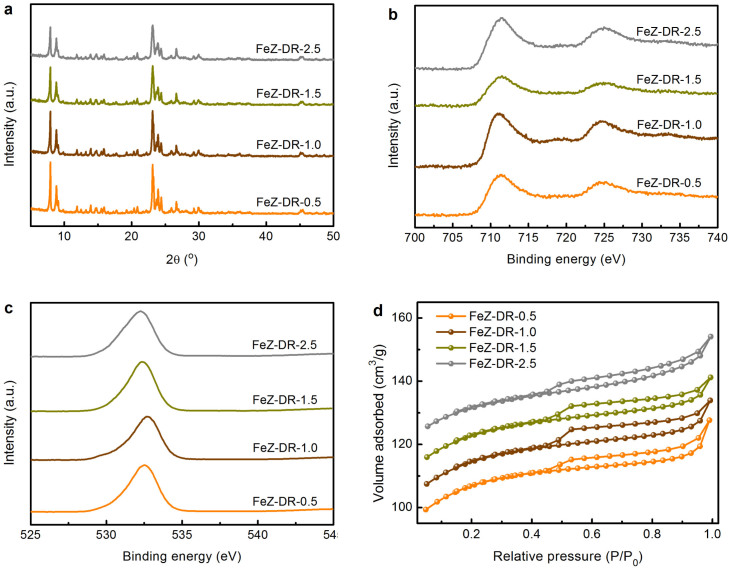
Characterizations of the different FeZSM-5 zeolites. (a) XRD patterns, (b) Fe 2*p* and (c) O 1*s* XPS spectra, and (d) nitrogen adsorption-desorption isotherms of the different FeZSM-5-n zeolites, where n refers to the mass percentage of iron in solid products determined by XRF.

In order to demonstrate the universality of such a green method, a series of hierarchical FeZSM-5s with different iron contents and highly-dispersed iron species were successfully prepared through elaborately adjusting the feed ratios according to the available diatomites of various grades, as shown in Figure 7. From the XRD patterns in Figure 7a, all of the crystalline products give diffractions belonging to MFI-type structure and have no other unidentified phase, indicating that pure-phase FeZSM-5 zeolites with iron contents ranging from 0.5 to 2.5 wt% were obtained. In these samples, iron ions are in the trivalent oxidation state and no peak attributed to the oxygen in Fe_x_O_y_ is detected (Figure 7b and 7c), suggesting the excellent dispersion of iron species. In addition, the isotherms of these FeZSM-5 zeolites are all of type IV ones (Figure 7d), demonstrating the presence of mesopores in these samples. These characterization and analysis results show that hierarchical FeZSM-5s with adjustable iron contents and highly-dispersed iron species can be successfully synthesized from thermally activated diatomite and SMS depolymerized rectorite minerals.

### Catalytic performance

**Figure 8:**
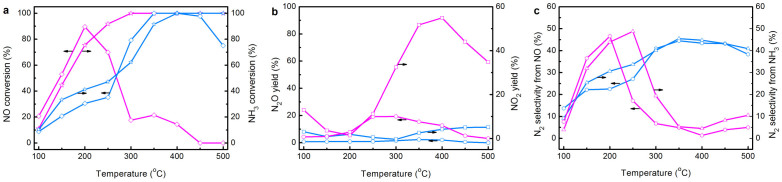
NH_3_-SCR of NO over FeZ-DR (blue) and FeZ-CA (magenta). (a) Conversion rates of NO and NH_3_, and (b) yields of N_2_O and NO_2_ and (c) selectivities of N_2_ from NO and NH_3_. Reaction conditions: 200 mg catalyst, 1000 ppm NO, 1000 ppm NH_3_, 6% O_2_ (balance He) and GHSV = 50000 h^−1^.

To explore the industrial potential of FeZ-DR, the NH_3_-SCR of NO over FeZ-DR and FeZ-CA was carried out in a microcatalytic flow reactor. The temperature dependence of the conversion of NO and NH_3_ into N_2_ over them is shown in Figure 8. It is clearly seen that FeZ-DR is very active for the SCR of NO with NH_3_, and the NO conversion obtained over FeZ-DR was dramatically higher than over FeZ-CA except in the low temperature region (≤250°C). Significantly, the temperature window for high NO conversion over FeZ-DR is notably wider than that over FeZ-CA, i.e., nearly 100% NO conversion can be maintained between 350 and 450°C. Remarkably, the main reaction product is N_2_, with almost no N_2_O or NO_2_ being detected over FeZ-DR in the whole temperature range (Figure 8b). In comparison, FeZ-CA showed lower activity for NO reduction under the same conditions, and the NO conversion decreased sharply when the temperature was >250°C due to oxidation of NH_3_ by oxygen^15^,^19^. Not surprisingly, the NH_3_ consumption over FeZ-CA was much higher than that over FeZ-DR below 400°C above which the NH_3_ consumption reached at the same level and the selectivities of N_2_ from NO and NH_3_ were also lower (Figure 8c), indicating that the NH_3_ utilization over FeZ-CA is poorer. Moreover, the undesired products (N_2_O and NO_2_) were relatively more over FeZ-CA than over FeZ-DR. The SCR activity of FeZ-DR and FeZ-CA in terms of turnover frequency (TOF) values is compared in Table 1^53^. It is clearly seen that the TOF of FeZ-DR is 4.5 times higher than that of FeZ-CA. Compared with the results in the literature, the activity of FeZ-DR is comparable to that of the FeZSM-5 with higher Fe loading (3.5 wt%) obtained by a more complicated and time-consuming method as reported by Shi *et al.*^3^ (whose TOF is 4.3 × 10^−3^ s^−1^), and also higher than that of the FeZSM-5 with similar Fe content obtained by Samaza *et al.*^21^ (whose TOF is 2.4 × 10^−3^ s^−1^). Why does FeZ-DR exhibit superior catalytic activity? This is because: (1) it has more isolated Fe^3+^ species (Table 2 and Supplementary Figure S8) that are active sites with high efficiency for NH_3_-SCR^20^,^45^; (2) it has a hierarchical micro-mesoporous structure that can improve the accessibility of active sites, accelerate the diffusion and enhance the internal mass transfer^29^,^47^.

## Conclusion

In summary, an economic and environmental benign strategy for synthesizing hierarchical FeZSM-5s via the nanoscale depolymerization-reorganization of natural aluminosilicate minerals is developed and a series of hierarchical FeZSM-5s with iron content of 0.5 ~ 2.5 wt% have been successfully synthesized. Our strategy is featured by neither using any aluminum-, silicon-, or iron-containing inorganic chemical nor involving any mesoscale template and any post-synthetic modification. More importantly, the resulting FeZSM-5s own hierarchical pore structure with mesopores of size 10 ~ 50 nm and outstanding dispersion of iron, and thus exhibit superior catalytic performance in NH_3_-SCR of NO, e.g. nearly 100% NO conversion can be maintained between 350 and 450°C and almost no N_2_O or NO_2_ is generated. This demonstrates a great perspective in environmental catalysis for reactions such as N_2_O direct decomposition, oxidation dehydrogenation of alkanes and selective hydroxylation of benzene to phenol. The nanoscale depolymerization-reorganization methodology can be easily extended to the preparation of other iron-containing zeolites such as iron-mordenite and iron-beta.

## Methods

### Materials

The natural rectorite mineral (43.2 wt% SiO_2_, 37.2 wt% Al_2_O_3_ and 0.5 wt% Fe_2_O_3_) used in the present study was purchased from Hubei Celebrities Rectorite Technology Company, Ltd. (Hubei Province, P. R. China). The natural diatomite minerals of different grades (85 ~ 96 wt% SiO_2_, 2.0 ~ 4.5 wt% Al_2_O_3_ and 0.5 ~ 5.0 wt% Fe_2_O_3_) were purchased from Qingdao Chuanyi Diatomite Company Ltd. (Shandong Province, P. R. China). Both of the two minerals were used as received without any further purification. NaOH and TPABr were purchased from the market.

### Depolymerization of the natural minerals

Typically, the natural diatomite mineral was treated by calcination at 600°C for 4 h in a muffle furnace with air circulation. The natural rectorite was treated as follows: the raw rectorite, NaOH and deionized water were mixed in an open Teflon drum, then the resulting rectorite-NaOH-H_2_O mixture was put into an oven exposed to air at 250°C for 2 h to perform the SMS depolymerization.

### Synthesis of FeZSM-5 zeolites

In a typical synthesis, the thermally activated diatomite was mixed with TPABr, SMS depolymerized rectorite, NaOH and deionized water under vigorous stirring with the final molar composition of the mixture at Al_2_O_3_:Fe_2_O_3_:SiO_2_:Na_2_O:TPABr:H_2_O = 1.1:0.26:40:6:4:1600. Subsequently, the resultant mixture was hydrothermally crystallized in a Teflon-lined stainless-steel autoclave at 170°C and autogenous pressure for 48 h. The thus-obtained solid product was recovered by filtration, washing with deionized water, and drying at 120°C overnight. The as-synthesized NaZSM-5 zeolite was calcined at 550°C for 6 h in air to decompose the organic template, and then converted to HZSM-5 by successive ion exchanges with a 1.0 M NH_4_Cl solution and calcinations at 520°C for 4 h.

### Characterizations

The Si, Al and Fe contents of the solid samples were determined by XRF conducted on a Bruker S4 Explorer instrument. The concentrations of silica, alumina and ferric oxide in the mother liquor at different crystallization times were quantified by inductively coupled plasma-atomic emission spectrometry (ICP-AES). XRD patterns of the samples were obtained on a Bruker AXS D8 Advance X-ray diffractometer with monochromatized Cu Kα radiation (40 kV, 40 mA). FTIR spectra were recorded on a Nicolet Magna-IR 560 ESP spectrometer (USA) using KBr discs at room temperature with 32 scans and 1 cm^−1^ resolution for each spectrum. The relative crystallinity of the samples was estimated from (I_550_/I_450_)/0.72 × 100%, with I_550_ and I_450_ being the intensities of the infrared bands near 550 and 450 cm^−1^ 28,35,37. These bands are related to the characteristic vibration of the double-five ring in MFI zeolite and the Si-O vibration, respectively^36^,^37^,^38^. The textural properties of the samples were examined by N_2_ adsorption-desorption experiments at −196°C on a Micromeritics ASAP 2420 instrument. Specific surface areas of the samples were calculated by the BET method, while the external surface areas and micropore volumes (V_micro_) were estimated using the de Boer t-plot method. The FESEM images of the samples were obtained on a field-emission environmental scanning electron microscope (FEI Quanta 200F). TEM images were taken using a FEI Tecnai F20 (200 kV) high resolution transmission electron microscope with the sample mounted onto a C-flat TEM grid. UV-visible spectra were measured on a Shimadzu UV-2550 (Japan) spectrometer in air against BaSO_4_. Deconvolution of the UV-visible spectra into individual bands was performed by a standard peak-fitting software. XPS characterization was performed on a Thermo Scientific K-Alpha instrument with a beam size of 400 μm. H_2_-TPR spectra were recorded with a home-made apparatus equipped with a thermal conductivity detector. Prior to the H_2_-TPR analysis, the FeZSM-5 zeolites were heated from room temperature to 600°C at a rate of 10°C/min and then cooled down to 70°C in a pure argon flow. The reduction of the FeZSM-5 zeolites by hydrogen was carried out in 30 mL/min flow of 10% H_2_ in argon at a linear heating rate of 10°C/min up to 1050°C.

### Catalytic tests

The NH_3_-SCR of NO was carried out in a catalytic micro-flow quartz reactor at temperatures between 100 and 500°C. Before each catalytic run, 200 mg of a catalyst were heated in flowing He to 500°C and maintained at this temperature for 30 min; after that, a feed mixture of 1000 ppm NO, 1000 ppm NH_3_, and 6% O_2_ in He was directed into the catalyst bed at a gaseous hourly space velocity (GHSV) of 50000 h^−1^. The composition of the effluent gas was analyzed by photometric devices for the detection of NO, NO_2_ and NH_3_, and N_2_O was determined by a mass spectrometry.

## Author Contributions

Y.Y. conducted all of the synthesis, characterizations and catalytic tests. P.Y., H.L. and C.Y. participated in the synthesis and characterization. X.B. initiated and guided this work.

## Supplementary Material

Supplementary Information Supplementary Information

## Figures and Tables

**Figure 1:**
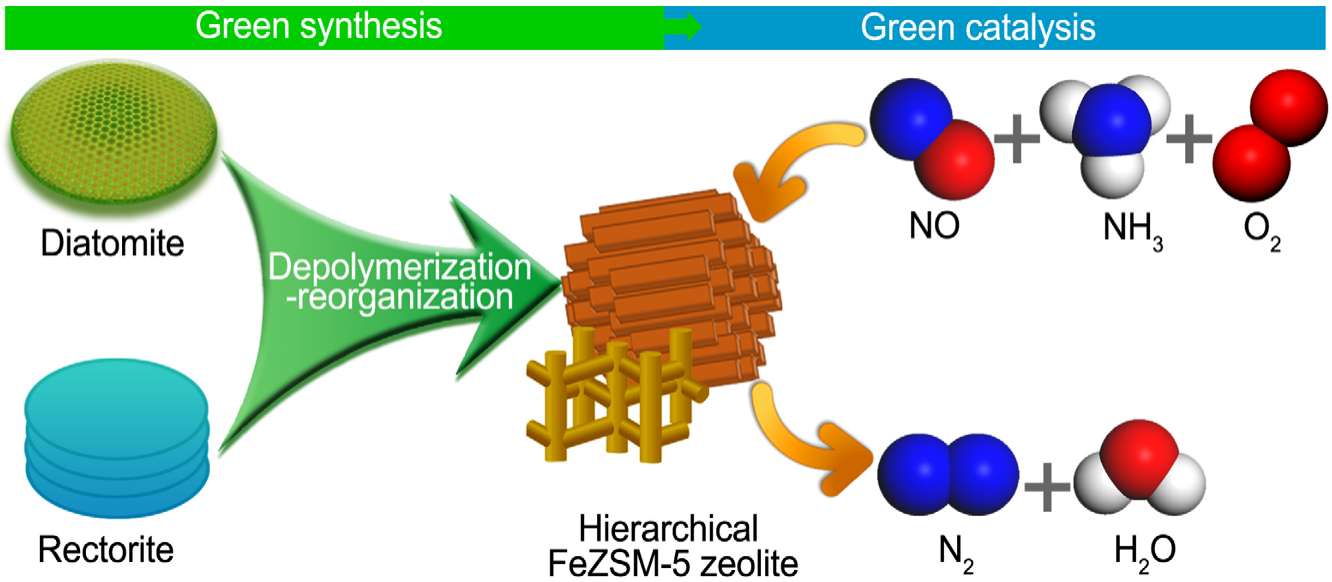
Schematic illustrating the one-pot synthesis of hierarchical FeZSM-5s and their application for NH_3_-SCR of NO.

**Table 1:** Data of FeZ-DR and FeZ-CA

Sample	SiO_2_/Al_2_O_3_ molar ratio^a^	Fe^a^ (wt%)	S_BET_ (m^2^/g)	S_micro_ (m^2^/g)	V_micro_ (cm^3^/g)	V_meso_ (cm^3^/g)	TOF^b^ × 10^3^ (s^−1^)
FeZ-DR	38.1	2.06	293	225	0.11	0.08	4.5
FeZ-CA	36.5	2.03	268	218	0.11	0.06	1.0

Notes:

^a^Determined by X-ray fluorescence (XRF).

^b^TOF is defined as the number of NO molecules converted per Fe per second (based on total Fe content) at the temperature of 300°C.

**Table 2:** Percentages of different iron species calculated from the deconvolution of UV-visible absorbance spectra of FeZ-DR and FeZ-CA

	Fe^a^		Fe^b^		Fe^c^		Fe^d^	
Catalyst	Center (nm)	Area (%)	Center (nm)	Area (%)	Center (nm)	Area (%)	Center (nm)	Area (%)
FeZ-DR	225	57	301	25	410	18	-	-
FeZ-DR^e^	226	52	300	29	406	19	-	-
FeZ-CA	220	42	300	22	410	24	521	12
FeZ-CA^e^	221	40	305	24	411	24	522	12

Notes:

^a^Isolated iron species in tetrahedral coordination.

^b^Isolated and oligonuclear Fe clusters.

^c^Large Fe clusters.

^d^Larger Fe_x_O_y_ particles.

^e^The sample treated at 500°C in He.
