# Supplementary material for: One-pot synthesis of hierarchical FeZSM-5 zeolites from natural aluminosilicates for selective catalytic reduction of NO by NH3
Source: Sci Rep. 2015 Mar 20;5:9270. doi: 10.1038/srep09270 (PMC4366855; doi:10.1038/srep09270)
Supplement: Supplementary Information [file srep09270-s1.pdf]

Supplementary Information:

One-pot synthesis of hierarchical FeZSM-5 zeolites from natural aluminosilicates for selective catalytic reduction of NO by NH_3

Yuanyuan Yue[1], Haiyan Liu[2], Pei Yuan[1]*, Chengzhong Yu[3], Xiaojun Bao[2]*

[1]*State Key Laboratory of Heavy Oil Processing, China University of Petroleum, Beijing 102249, P. R. China.*

[2]*The Key Laboratory of Catalysis, China National Petroleum Corporation, China University of Petroleum, Beijing 102249, P. R. China.*

[3]*Australian Institute for Bioengineering and Nanotechnology, the University of Queensland, Brisbane St Lucia, QLD 4072, Australia.*

**Correspondence to: to whom correspondences and requests for materials should be addressed. E-mail: yuanpei@cup.edu.cn(P. Yuan); baoxj@cup.edu.cn(X. Bao).*

Supplementary figures

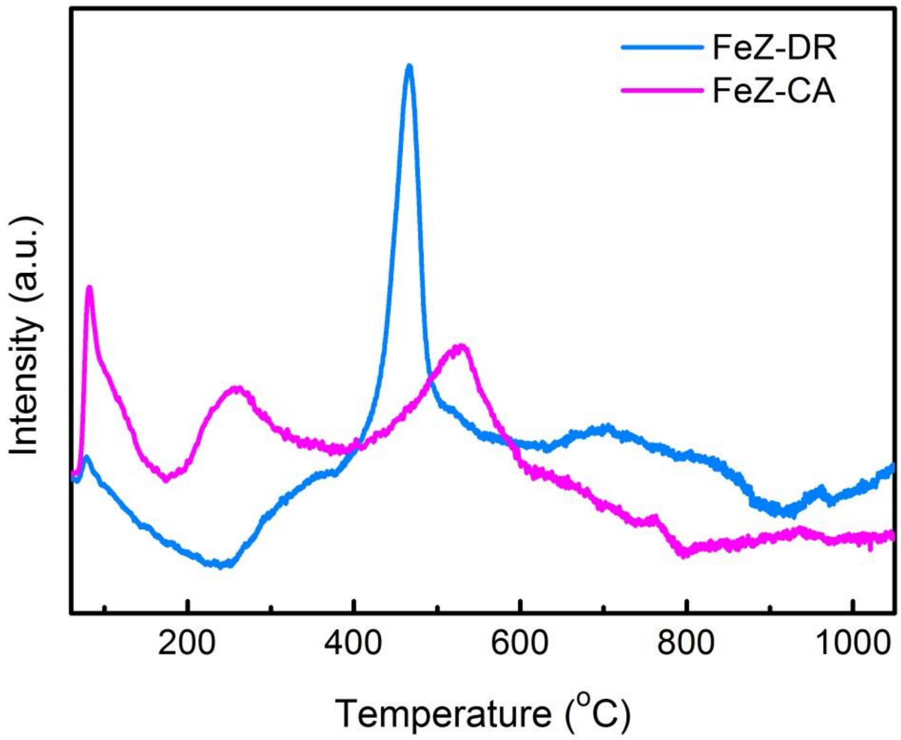

Supplementary Figure S1. H_2-TPR profiles of FeZ-DR and FeZ-CA. Both profiles show the broad peak centered at *ca*. 730 °C attributed to framework Fe (III) that is hard to reduce, suggesting that the Fe^{3+} species are incorporated into the zeolite framework in tetrahedral coordination[1,2].

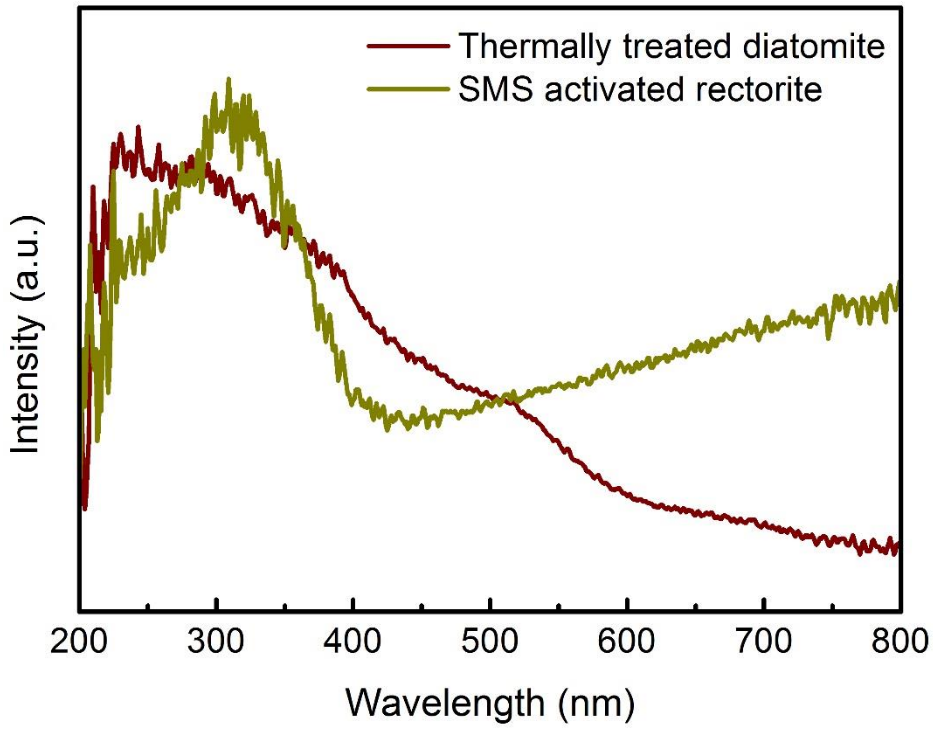

Supplementary Figure S2. UV-visible spectra of the thermally activated diatomite and SMS depolymerized rectorite. It is clearly seen that both spectra have the absorbance bands at *ca.* 225 and 320 nm attributed to oxygen-to-iron p-d and d-d charge transfers[3], respectively. This demonstrates that the treated minerals both contain isolated Fe^{3+} in tetrahedral coordination and bi- and oligonuclear Fe clusters.

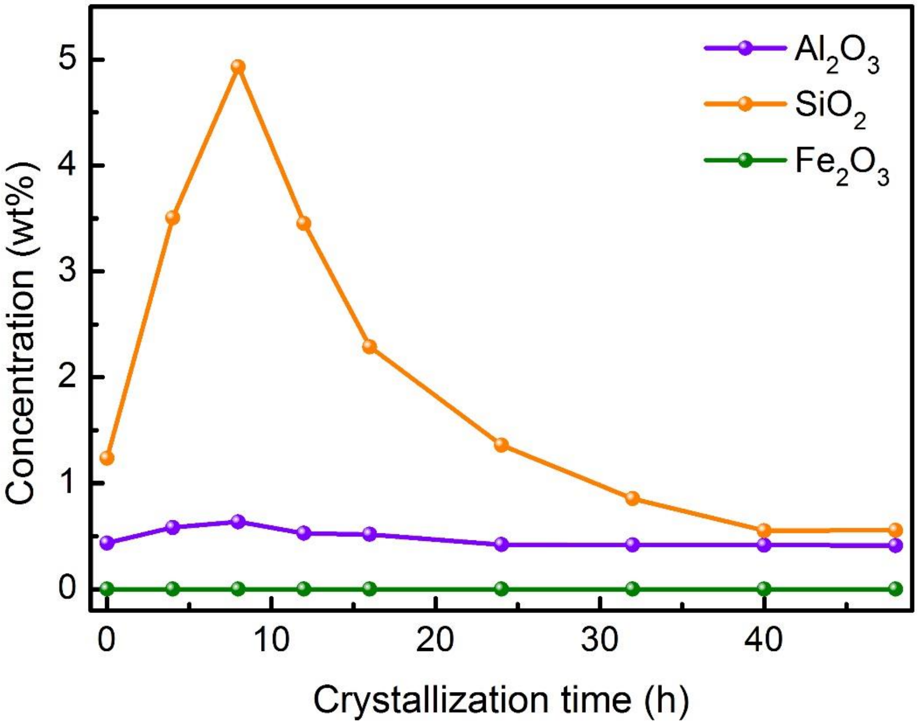

Supplementary Figure S3. Concentrations of silica, alumina and ferric oxide in the mother liquor at different crystallization times.

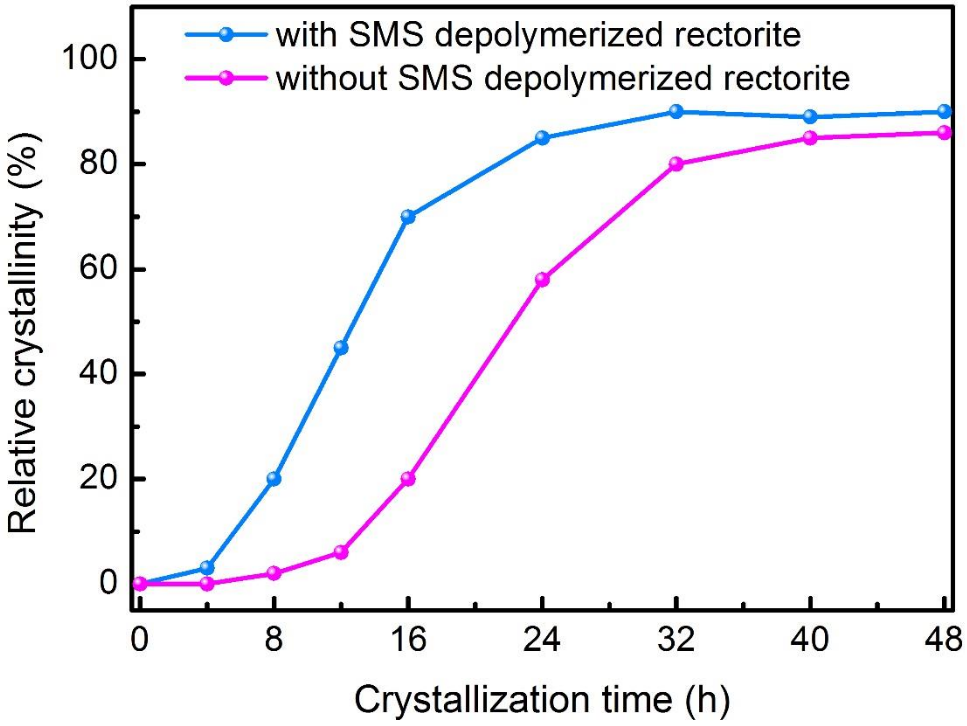

Supplementary Figure S4. Crystallization curves of the FeZSM-5 zeolites obtained with and without using the SMS depolymerized rectorite, respectively.

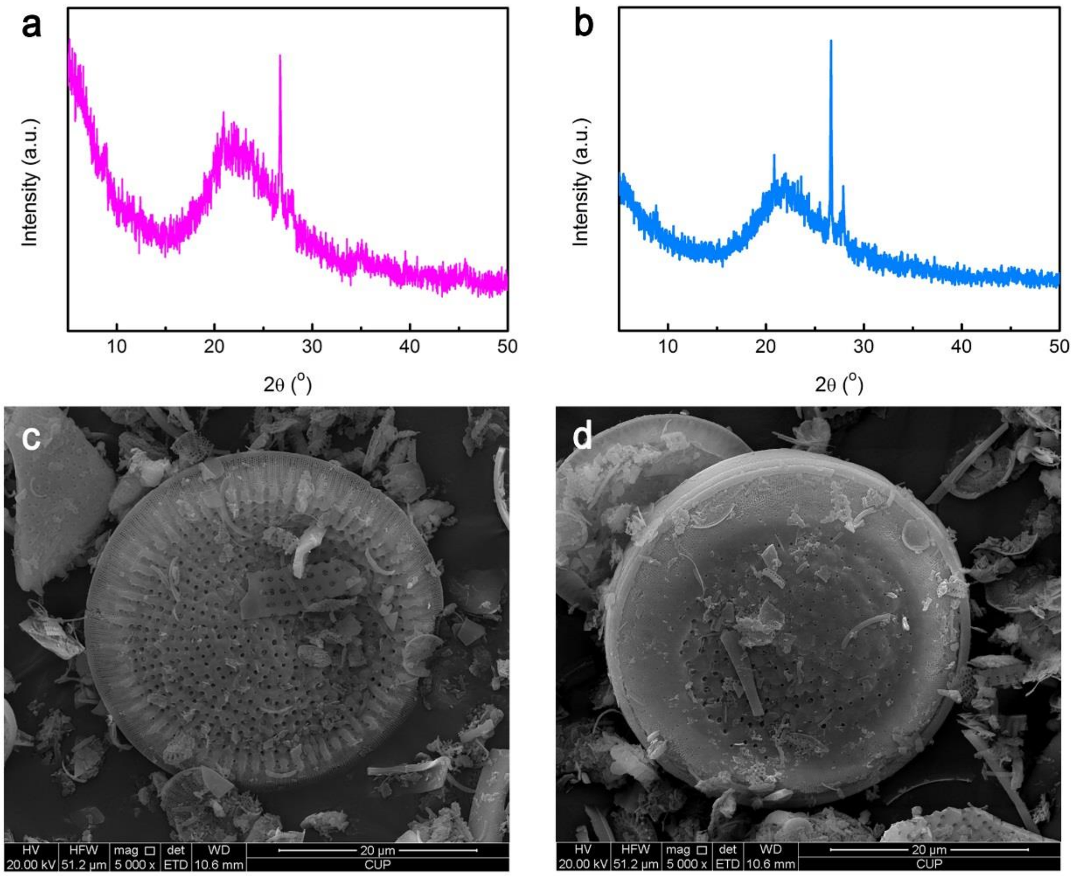

Supplementary Figure S5. XRD patterns and FESEM images of the solid samples obtained using the thermally activated diatomite and aluminum sulfate as silicon, aluminum and iron sources at different crystallization times: 0 h (**a**, **c**) and 48 h (**b**, **d**). Obviously, an aluminosilicate gel overlays the outer surface of diatomite and impedes its further dissolution, leading to the formation of an amorphous solid after crystallization for 48 h.

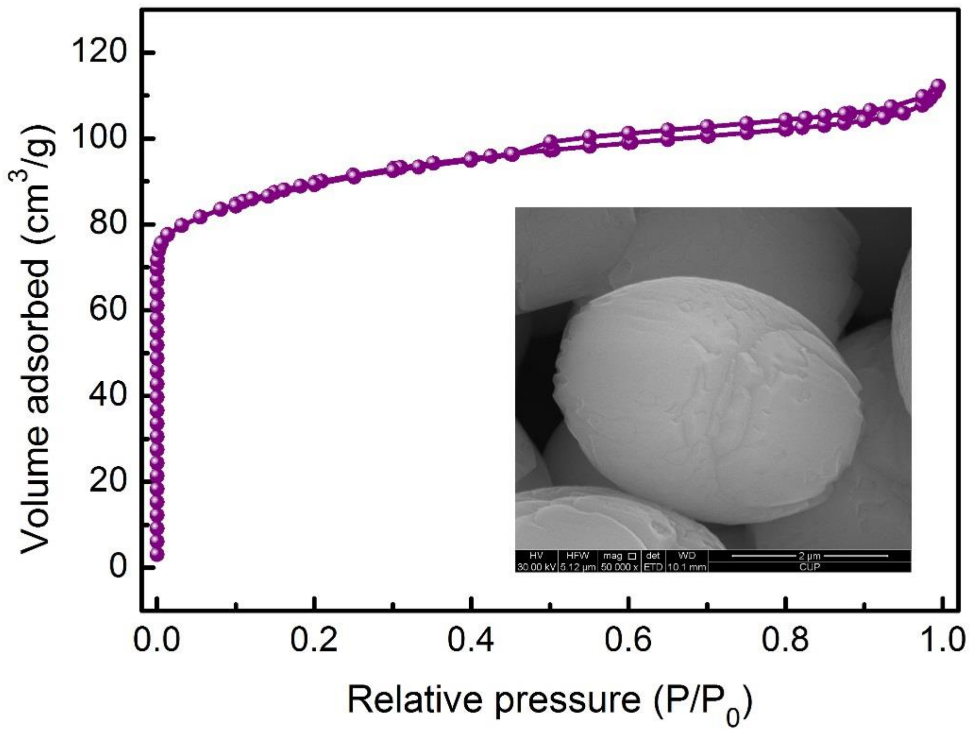

Supplementary Figure S6. Nitrogen adsorption-desorption isotherms and FESEM images (inset) of the solid samples obtained using sodium silicate and the SMS depolymerized rectorite as the silicon, aluminum and iron sources.

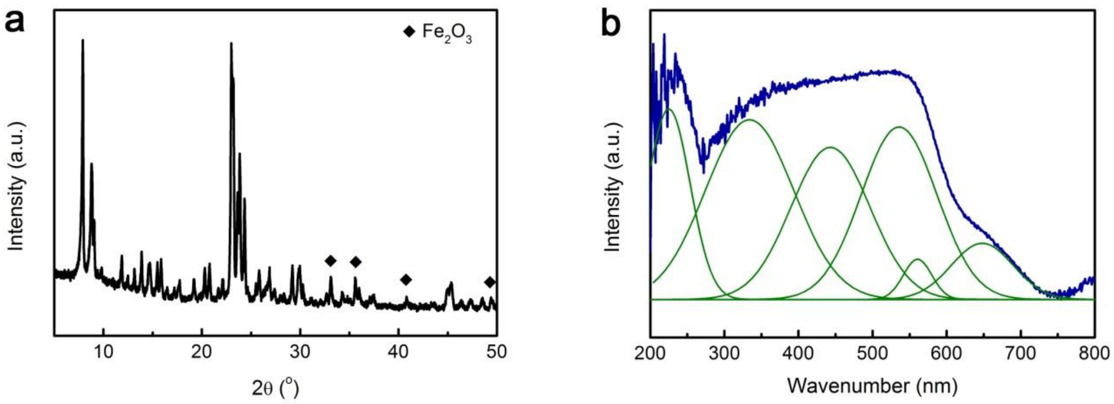

Supplementary Figure S7. (**a**) XRD pattern and (**b**) UV-visible spectra of the FeZSM-5 zeolite synthesized by using water glass, sodium aluminate, ferric oxide and TPABr as silicon, alumina and iron sources and template, respectively. The typical XRD peaks of Fe_2O_3 can be observed in the pattern at 2θ of 33.1°, 35.6°, 40.8° and 49.5° [4,5], and the UV-visible absorbance peaks of Fe_2O_3 particles also exist above 450 nm. This suggests that only a small amount of iron have been incorporate into the zeolite framework, although the solubility of the ferric oxide used as the iron source is low in the alkaline system. By combining the analysis results in the text body, we can certainly draw a conclusion that the iron species in the minerals are transformed in situ into the zeolite framework.

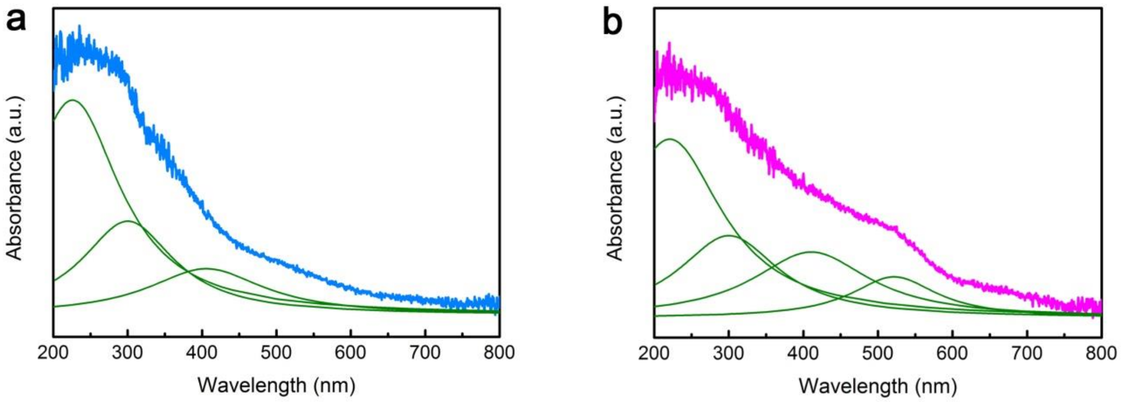

Supplementary Figure S8. UV-visible spectra of (**a**) FeZ-DR and (**b**) FeZ-CA after treated at 500 ^{o}C in He.

Supplementary references

1 Pérez-Ramírez, J. *et al.* Physicochemical characterization of isomorphously substituted FeZSM-5 during activation. *J. Catal.* **207**, 113-126, (2002).

2 Bordiga, S. *et al.* Structure and reactivity of framework and extraframework iron in Fe-silicalite as investigated by spectroscopic and physicochemical methods. *J. Catal.* **158**, 486-501, (1996).

3 Gu, J. *et al.* Unseeded organotemplate-free hydrothermal synthesis of heteroatomic MFI zeolite poly-nanocrystallites. *J. Mater. Chem. A* **1**, 2453-2460, (2013).

4 Iwasaki, M., Yamazaki, K., Banno, K. & Shinjoh, H. Characterization of Fe/ZSM-5 $DeNO_x$ catalysts prepared by different methods: Relationships between active Fe sites and NH_3-SCR performance. *J. Catal.* **260**, 205-216, (2008).

5 Shi, X., Liu, F., Xie, L., Shan, W. & He, H. NH_3-SCR performance of fresh and hydrothermally aged Fe-ZSM-5 in standard and fast selective catalytic reduction reactions. *Environ. Sci. Technol.* **47**, 3293-3298, (2013).
